# Impact of vitamin D deficiency on clinical outcomes in non-traumatic subarachnoid hemorrhage: A single-center prospective cohort study

**DOI:** 10.1038/s41598-026-38728-9

**Published:** 2026-02-04

**Authors:** Dorottya Szántó, Béla Fülesdi, Lili Simon, László Novák, János Kappelmayer, Csilla Molnár

**Affiliations:** 1 Department of Anesthesiology and Intensive Care, Nagyerdei krt. 98., H-4032 Debrecen, Hungary; 2 Department of Neurosurgery, Debrecen, Hungary; 3 Department of Laboratory Medicine, Faculty of Medicine, University of Debrecen, Debrecen, Hungary

**Keywords:** Vitamin d, Subarachnoid hemorrhage, Inflammation, Brain ischemia, Neuroprotection, Infections, Biomarkers, Diseases, Medical research, Neurology, Neuroscience

## Abstract

There is growing evidence that neuroinflammation and systemic inflammatory response following SAH have a major effect on patients’ outcomes. We hypothesized that due to the immunomodulating and neuroprotective properties, Vitamin D deficiency may contribute to unfavorable outcome in SAH patients. Consecutive patients admitted with acute non-traumatic SAH were prospectively included. Within 24 h of admission, serum 25(OH)vitamin D levels were measured. During the first 21 days after SAH, inflammatory markers were closely monitored, the development of any infectious complication was noted, and the onset of cerebral vasospasm was evaluated using transcranial color-coded Doppler. New ischemic lesions on follow-up computed tomography imaging were also documented. Patients’ outcomes were assessed according to the modified Rankin Scale (mRS) and the Barthel’s Index on days 14, 30, and 90. Onehundred fifteen patients were included, of whom 61 were vitamin D deficient (VDD, serum vitamin D < 50 nmol/L) and 54 were vitamin D sufficient (VDS, serum vitamin D ≧ 50 nmol/L). The incidence of new ischemic lesions was significantly higher in the VDD group (VDD: 68.63% vs. VDS: 40.74%, *p* = 0.008). The VDD group also had higher peaks in inflammatory markers and a tendency of higher incidence of infections (VDD: 42.62% vs. VDS: 24.07%, *p* = 0.057). A poor outcome, as defined by the mRS, was significantly more frequent among VDD patients (on day 90: VDD, 61.02% vs. VDS, 35.19%, *p* = 0.011). Our findings raise the potential harmful effects of D hypovitaminosis among patients with SAH. Vitamin D deficiency may contribute to greater inflammatory response and increased risk of secondary ischemia, infections, and poor clinical outcomes. Further studies will show whether low vitamin D levels measured at admission are the consequence of SAH severity or whether pre-ictus hypovitaminosis also plays a role in determining the worse prognosis.

**Supplementary Information:**

The online version contains supplementary material available at 10.1038/s41598-026-38728-9.

## Introduction

Despite advances in endovascular techniques and neurocritical care, acute, non-traumatic subarachnoid hemorrhage (SAH) still has a high morbidity and mortality rate. Roughly half of the survivors have significant disability and are unable to return to their previous lives. Besides early injury, the development of delayed cerebral ischemia (DCI) or certain systemic complications may significantly influence patients’ outcomes^1–3^. Based on recent data, both neuroinflammation and systemic inflammation play a prominent role in the pathomechanism of major complications following SAH^4,5^.

SAH induces a systemic inflammatory response that contributes to the development of a wide range of systemic complications (e.g., Takotsubo cardiomyopathy and neurogenic pulmonary edema) and amplifies neuronal loss^4,6,7^. The injured nervous tissue and components of the degrading hematoma also activate the immune system by inducing the expression of proinflammatory cytokines and recruiting immune cells into the site of the injury. This process is essential for hematoma clearance. Nevertheless, neuroinflammation also contributes to secondary brain injury after SAH^8,9^.

Based on these, experimental and clinical studies drew attention to anti-inflammatory and immunomodulatory agents, which may influence the inflammatory response and contribute to recovery. However, none of them has been proven to be effective, and most of these agents are associated with multiple side effects, some of which may be severe^10^. –^11^.

Recently, Vitamin D is receiving increasing attention due to its neuroprotective, immunomodulatory, and antimicrobial properties (12). Vitamin D acts as a secosteroid hormone, exerting pleiotropic effects in the body. Besides its well-known effect on bone metabolism, it has immunomodulatory properties, and it also exerts effects on cell differentiation and proliferation ^12–16^. It promotes antiinflammatory cytokine production and the development of antiinflammatory immune cells, whereas it suppresses proinflammatory pathways^13,17^. Vitamin D shows a negative correlation with several inflammatory markers in a wide range of clinical conditions, such as autoimmune, cardiovascular, and infectious diseases^18–21^.

The role of vitamin D in SAH is yet less well explored, and prospective studies investigating the effect of vitamin D on SAH complications and outcome are missing. Based on this, the authors aimed to investigate whether vitamin D deficiency contributes to the development of certain neurological and non-neurological complications and worse clinical outcomes after SAH.

## Methods

### Patient population and study design

This prospective observational cohort study was conducted in our 8-bed neurosurgical intensive care unit of the University of Debrecen, Hungary, between June 2022 and February 2025. The study was approved by the Institutional Ethics Committee University of Debrecen (Approval number: DE KK RKEB/IKEB 5924/2021), and written informed consent was obtained from all patients or their legal representatives. The study was registered on 31 May 2022 at ClinicalTrials.gov with the registration number NCT05403970 (https://clinicaltrials.gov/study/NCT05403970?term=NCT05403970&rank=1&tab=history). All methods were performed in accordance with the relevant guidelines and regulations.

We applied the following inclusion criteria: acute non-traumatic SAH (including aneurysmal SAH, aneurysmal-pattern SAH without angiographically confirmed aneurysm, and perimesencephalic SAH), adult age (≥ 18 years), and hospital admission within 48 h of symptom onset. Patients with SAH secondary to head trauma, angioma, or arteriovenous malformations were excluded. All participants or their legal representatives provided written informed consent prior to participation.

On admission, clinical and radiological severity of SAH was evaluated according to World Federation of Neurological Society (WFNS) score, Hunt–Hess score, Glasgow Coma Scale (GCS), and modified Fisher grade. Decision on tratment modality (cliping or coiling) was made in every case based a consultation between neurosurgeons and interventional neuroradiologists. Demographic characteristics and medical history of patients were also recorded. Moreover, blood sampling was performed to measure routine laboratory parameters, inflammatory markers, and serum 25-hydroxyvitamin D [25(OH)D] levels.

According to serum 25(OH)D levels, we stratified our patients into two groups, based on Endocrine Society guidelines^22^:

- Vitamin-D deficient group – VDD: < 50 nmol/L (< 20 ng/mL).
- Vitamin-D sufficient group - VDS : ≥ 50 nmol/L (≥ 20 ng/mL).

During the first 21 days following SAH, the development of vasospasm (a), newly emerging ischemic lesions on CT imaging (b), and the development of any infectious complications (c) were noted; inflammatory markers were closely monitored (d).

- Cerebral vasospasm was diagnosed by transcranial color-coded duplex sonography (TCCD). TCCD was performed by experienced investigators, using a 2 MHz sector transducer on the GE Venue Go ultrasound system (GE Healthcare 9900, Innovation Drive, Wauwatosa, WI, USA). Insonation was conducted through the transtemporal acoustic window. Bilateral daily measurements were obtained from the middle cerebral, anterior cerebral, and posterior cerebral arteries. According to previously published criteria^23^, vasospasm was defined as a mean blood flow velocity exceeding 120 cm/s. All TCCD measurements were performed by a neurologist/neurosonologist (BF), having several decades of experience. Digital subtraction angiography (DSA) or CT angiography (CTA) was performed in cases where TCCD referred to severe vasospasm or in those cases where TCCD was not feasible due to an inappropriate window, but clinical signs referred to probable vasospasm. Finally, 27 patients underwent DSA, and 5 patients underwent CTA assessment.
- Delayed cerebral ischemia was defined as the combination of worsening the clinical signs and new ischemic lesions on the image. New ischemic lesions were assessed using native cranial CT imaging. Cranial CT was performed if it was clinically indicated (focal neurological deficits, worsening of GCS by ≥ 2 points lasting for at least 60 min). Cranial CTs were reviewed by experienced neuroradiologists in all cases, who were blinded to patients outcomes.
- Clinically significant infections — including pneumonia, meningitis, urinary tract infections, bloodstream infection, and sepsis — were diagnosed based on the treating physician’s judgment, supported by the patient’s symptoms, laboratory markers, results of microbiological testing, and imaging findings. Based on the presence or absence of any type of infection, we categorized our patients into „infection positive” (Inf+) and „infection negative” (Inf-) groups.
- Serum IL-6 (as a fast-reacting proinflammatory cytokine) and C-reactive protein (marker with a more delayed peak but sustained elevation) levels, and blood leukocyte count and composition were measured at least every two days or more frequently, as it was clinically indicated. We also calculated the neutrophil-to-lymphocyte ratio (NLR), as it reflects both inflammation driven by neutrophils and immune suppression related to low lymphocyte levels, offering a more balanced view of the immune response.

Neurointensive care management strategies:

- Blood pressure target strategy: The institutional protocol is based on recent recommendations^24^. Accordingly, systolic blood pressure was kept below 160 mmHg before the treatment of the aneurysm, and hypotension was avoided. For keeping the optimal target blood pressure after aneurysm closure, our institutional protocol involves invasive blood pressure monitoring. If the patient’s pre-ictus blood pressure is known, the goal is to maintain blood pressure at that level and prevent hypotension. If a severe vasospasm developed, our strategy was hemodynamic augmentation. Selecting the optimal vasopressor depended on the patient’s cardiovascular status and the severity of the vasospasm. Most commonly, norepinephrine was used, eventually supplemented by positive inotropes, such as dobutamine. In case of severe vasospasm, the augmentation of the cerebral blood flow is far more important; for this purpose, milrinone therapy without a loading dose was used to prevent hypotension. The upper systolic blood pressure value was not allowed to exceed 200 mmHg.
- Euvolemia strategy: The local protocol prescribes maintenance of strict volume control throughout the entire duration of treatment to prevent both hypovolemia and hypervolemia. Fluid balance was assessed on an hourly basis. Hypotensive episodes were corrected by using vasopressors like noradrenaline.
- Sodium management: According to our institutional protocol, correction of serum sodium concentration started when serum sodium level falls below 130 mEq/L. During the correction procedure, euvolemia was maintained, and the correction of hyponatremia occurred by administration of hypertonic (3%) NaCl slowly. If the hyponatremia was resistant to sodium chloride administration, mineralocorticosteroid therapy (fludrocortisone) was used. In all cases, the patient’s medication was revised and medications potentially causing or worsening hyponatremia were corrected.

Severe hypernatremia was diagnosed when the sodium level exceeded 155 mmol/L. The treatment strategy started with the replacement of fluid, enteral free water via nasogastric tube, or intravenous fluid replacement. If the cause was identified as diabetes insipidus (DI), the treatment strategy included desmopressin (DDAVP) as hormonal replacement.

- Oral nimodipine 6 × 60 mg was a routine part of the treatment strategy for the first 21 days after admission.
- Insertion and removal of EVD: Insertion of endovascular drain occurred in all cases after neurosurgical condultation. In patients with aSAH and acute symptomatic hydrocephalus, urgent EVD insertion was performed to improve neurological outcome. In patients with aSAH and associated chronic symptomatic hydrocephalus, permanent CSF diversion, EVD or LD insertion occurred according to the neurosurgeon’s indication. The decision was based on the patient status and TCCD data. (a) If the patient’s neurological status worsened by more than 2 points according to GCS score, as well as S/D was more than 3, or PI was more than 1.3 during TCCD measurement, drain insertion was indicated. Removal timing was based on clinical signs and ICP measurement data. Weaning from EVD was considered, if CSF was nonbloody; CSF output was ≤ 250 ml; and if ICP ICP ≤ 20–22 mmHg for 24–48 h, the drain was removed.

Functional outcomes on days 14, 30, and 90 were assessed using the modified Rankin Scale (mRS) and Barthel Index (BI). An unfavourable outcome was defined as an mRS score > 2 or a Barthel Index score < 50. Mortality data were also recorded.

### Statistical analysis

For statistical analysis, the SPSS 29.0 (IBM Corp., Armonk, NY, USA) and R 4.5.0 statistical softwares were used. The homogeneity of variances was checked with Levene’s test, and the normal distribution of variables was checked by the Shapiro–Wilk test. Unpaired T-test was applied to compare normally distributed variables, which were reported as mean ± SD. For non-normally distributed variables and/or for those with heterogeneous variances, descriptive statistics were reported as median (interquartile range, IQR), and the Mann-Whitney U-test was used to compare the differences between two independent groups. Categorical variables were compared using the Chi-square test. When 2 × 2 tables were analysed, we applied Yates’ correction for continuity. When the number of expected cases was too small (< 5) in more than 20% of the cells, Fisher’s exact test was used. For 2 × 2 comparisons, alongside the χ² (or Fisher’s exact) p-values, unadjusted odds ratios (OR) with 95% confidence intervals (CI) were also reported. Receiver operating characteristic (ROC) curve analysis was performed to evaluate the association between vitamin D levels and outcome measures. The area under the curve and 95% confidence intervals were estimated. Risk-factor–outcome associations were evaluated using multivariable logistic regression. Adjusted odds ratios with 95% confidence intervals and two-sided p-values were reported. It was further examined whether the association between vitamin D deficiency and 90-day outcome (mRS90) was partly mediated by post-SAH complications. Two separate single-mediator analyses were run: one with new ischaemic lesions as the mediator and one with pneumonia as the mediator. The total effect (TE) of vitamin D deficiency on outcome was reported together with its decomposition into a direct effect (ADE – average direct effect) and an indirect (mediated) effect (ACME – average causal mediation effect) (Supplemental Fig. 1.). The two mediators were analysed separately to keep models simple and to preserve power given the modest sample size. *p* < 0.05 is considered statistically significant.

## Results

### Baseline characteristics

**Fig. 1:**
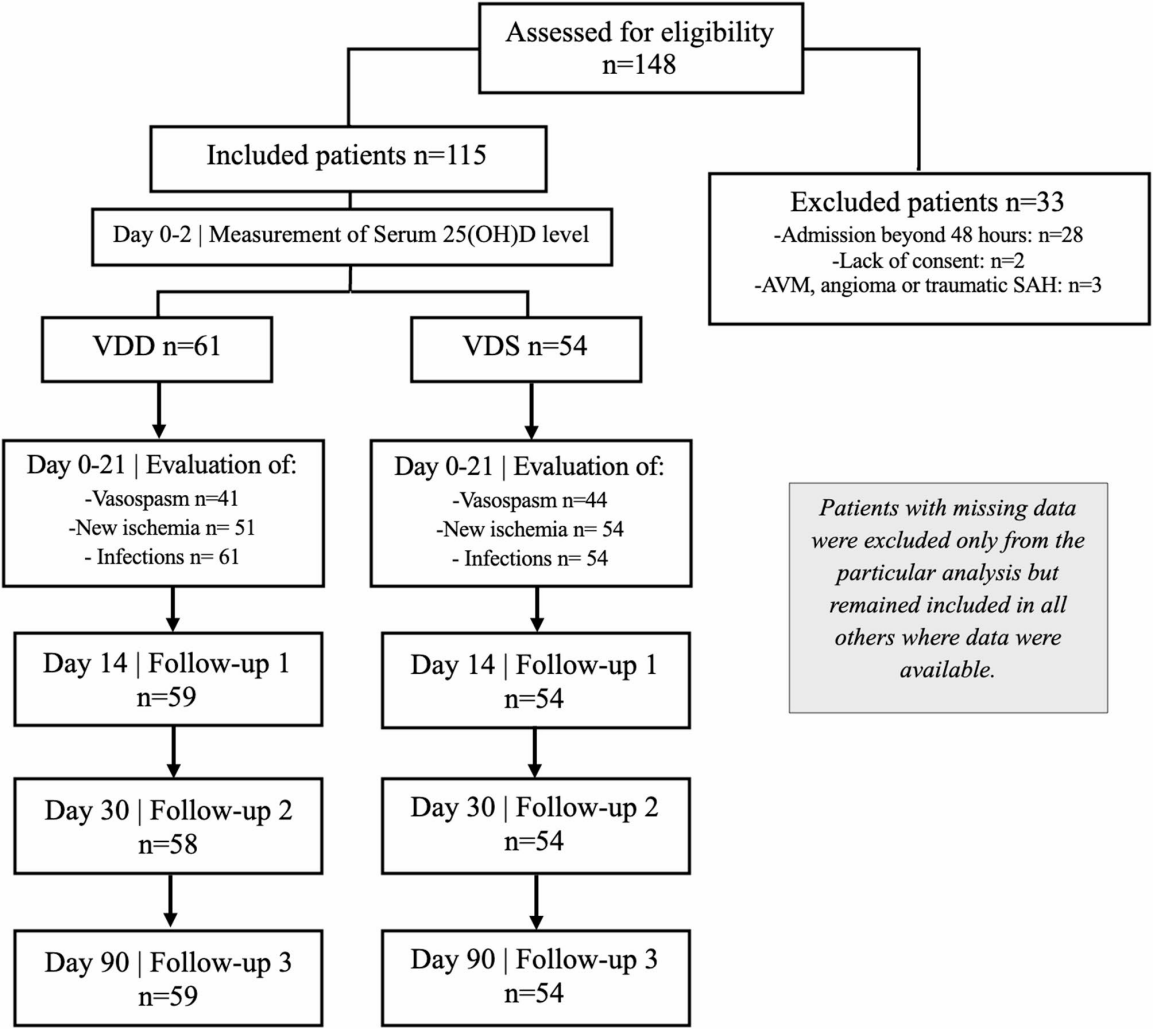
Flowchart of patient inclusion and exclusion. AVM indicates arteriovenous malformation.

A total of 115 patients with acute subarachnoid hemorrhage (SAH) were included in our study, among whom 74 (64.35%) were females and 41 (35.65%) were males, with an average age of 56.04 ± 12.68 years. Figure 1 shows the study design, including patient inclusion and exclusion, and the number of patients at each stage of the follow-up. Based on serum 25(OH)D levels at admission, patients were divided into two groups: VDD (< 50 nmol/L) and VDS (≥ 50 nmol/L). The VDD group included 61 patients (53%), while the VDS 54 patients (47%).

The most important clinical data of patients are summarized in Table 1. There was no significant difference in demographic characteristics, SAH severity scores, location of aneurysm, treatment modalities, chronic diseases, and chronic medication use between groups. However, aneurysmal SAH was significantly more frequent among VDD patients compared to VDS patients (90.16% vs. 66.67%, *p* = 0.004). In addition, the proportion of smokers was significantly higher in the VDD group (37.70% vs. 16.67%, *p* = 0.021).

**Table 1:** Baseline characteristics of the total cohort and comparison by vitamin D status. VDD: vitamin D deficient group. VDS: vitamin D sufficient group. IVH: intraventricular hemorrhage. ICH: intracerebral hemorrhage. WFNS: world federation of Neurosurgeons. GCS: Glasgow coma Scale. SAH: subaracnoid hemorrhage. AComA: anterior communicating artery. MCA: medial cerebral artery. BA: Basilar artery. COPD: chronic obstructive pulmonary disease. eGFR: estimated glomerular filtration rate. BMI: body mass index. ACEI/ATB: angiotensin-converting enzyme inhibitor/ angiotensin receptor blocker. OAD: oral antidiabetic drugs.

Parameters		Total Study Population (*n*/*N*, %)	Comparison of Study Groups Based on Vitamin-D Status		
			VDD (*n*/*N*, %)	VDS (*n*/*N*, %)	*p*-Value (Fisher/ χ^2^)
Number of patients (n)		115	61/115 (53%)	54/115 (47%)	
Gender (F)		74/115 (64.35%)	44/61(72.13%)	30/54 (55.56%)	*p* = 0.098
Age (years)		56.04 ± 12.68	57.7 ± 12.98	54.17 ± 12,31	*p* = 0.138
modified Fisher score (> 2)		86/115 (74.78%)	49/61 (80.33%)	37/54 (68.51%)	*p* = 0.215
IVH		63/115 (54.78%)	35/61 (57.38%)	28/54 (51.85%)	*p* = 0.684
ICH		28/115 (24.35%)	15/61 (24.59%)	13/54 (24.07%)	*p* > 0.999
Hunt-Hess score (> 3)		41/115 (35.65%)	25/61(40.98%)	16/54 (29.63%)	*p* = 0.283
WFNS score (> 3)		47/115 (40.87%)	29/61 (47.54%)	18/54 (33.33%)	*p* = 0.175
GCS (< 9)		38/115 (33.04%)	21/61 (34.43%)	17/54 (31.48%)	*p* = 0.891
Aneurysmal SAH		91/115 (79.13%)	55/61(90.16%)	36/54 (66.67%)	*p* = 0.004^*^
Aneurysm Location	AComA	29/91 (31.87%)	15/55 (27.27%)	14/36 (38.89%)	*p* = 0.351
	MCA	25/91 (27.47%)	17/55 (30.91%)	8/36 (22.22%)	*p* = 0.504
	ICA	17/91 (18.68%)	11/55 (20.0%)	6/36 (16.67%)	*p* = 0.788
	BA	8/91 (8.79%)	5/55 (9.09%)	3/36 (8.33%)	*p* > 0.999
	Other rare locations	12/91 (13.19%)	7/55 (12.73%)	5/36 (13.89%)	*p* > 0.999
Comorbidities and Risk Factors	Hypertension	64/115 (55.65%)	35/61 (57.38%)	29/54 (53.70%)	*p* = 0.835
	Diabetes mellitus	10/115 (8.69%)	4/61(6.56%)	6/54 (11.11%)	*p* = 0.512
	Ischaemic heart disease	10/115 **(8.70%)**	7/61 **(11.48%)**	2/54 **(3.70%)**	*p* = 0.170
	Valvular heart disease	2/115 **(1.74%)**	1/61**(1.64%)**	1/54 **(1.85%)**	*p* > 0.999
	Chronic or paroxysmal atrial fibrillation	3/115 **(2.61%)**	1/61**(1.64%)**	2/54 **(3.70%)**	*p* = 0.600
	Asthma or COPD	7/115 (6.09%)	4/61 (6.56%)	3/54 (5.56%)	*p* > 0.999
	Hypothyreosis	8/115 (6.96%)	3/61 (4.92%)	5/54 (9.26%)	*p* = 0.472
	Psychiatric disease	7/115 (6.09%)	4/61 (6.56%)	3/54 (5.56%)	*p* > 0.999
	Renal impairment	7/115 (6.09%)	4/61 (6.56%)	3/54 (5.56%)	*p* > 0.999
	- eGFR: 60–89 mL/min/1,73 m²	5/115 (4.35%)	3/61 (4.92%)	2/54 (3.70%)	*p* > 0.999
	- eGFR: 30–59 mL/min/1,73 m²	1/115 (0.87%)	1/61 (1.64%)	0/54 (0.00%)	*p* > 0.999
	- eGFR: <30 mL/min/1,73 m²	1/115 (0.87%)	0/61 (0.00%)	1/54 (1.85%)	*p* > 0.999
	Obesity (BMI ≥ 30)	34/115 (29.57%)	17/61 (27.87%)	17/54 (31.48%)	*p* = 0.827
	Alcohol abuse	6/115 (5.22%)	5/61 (8.2%)	1/54 (1.85%)	*p* = 0.212
	Smoking	32/115 (27.83%)	23/61 (37.70%)	9/54 (16.67%)	*p* = 0.021^*^
Medications Used Prior to Admission	ACEI/ARB	28/115 (24.35%)	13/61 (21.31%)	15/54 (27.78%)	*p* = 0.556
	Calcium-chanel blocker	14/115 (12.17%)	6/61 (9.84%)	8/54 (14.81%)	*p* = 0.569
	Beta-blocker	17/115 (14.78%)	9/61 (14.75%)	8/54 (14.81%)	*p* > 0.999
	Statin	11/115 (9.57%)	7/61 (11.48%)	4/54 (74.07%)	*p* = 0.537
	Antiplatelet medication	17/115 (14.78%)	11/61 (18.03%)	6/54 (11.11%)	*p* = 0.431
	OAD	6/115 (5.22%)	3/61 (4.92%)	3/54 (5.56%)	*p* > 0.999
	Diuretics	13/115 (11.3%)	7/61 (11.48%)	6/54 (11.11%)	*p* > 0.999

Table 2. summarizes the interventions in the different groups, including aneurysm treatment, placement of ventricular or lumbar drains. The frequency of surgical and endovascular interventions was similar between VDD and VDS patients; no differences reached statistical significance. In the total cohort, endovascular coiling was most common (~ 76%), surgical clipping occurred in ~ 16%, and ~ 8% received no aneurysm treatment, due to early mortality or aneurysm location precluding intervention. Ventricular drain placement was frequent (~ 45%; vs. ~43% within 72 h), and primary lumbar drains (not preceded by ventricular drainage) were uncommon (~ 7%). Intra-arterial spasmolysis occurred in ~ 20% (≥ 4 sessions in ~ 11%), with comparable rates in VDD versus VDS.

**Table 2:** Distribution of surgical and endovascular interventions after SAH dichotomized according to vitamin D status at admission.

Type of intervention	Total Study Population (*n*/*N*, %)	Comparison of Study Groups Based on Vitamin-D Status		
		VDD (*n*/*N*, %)	VDS (*n*/*N*, %)	*p*-Value (χ2)
Surgical clipping	15/91 (16.48%)	9/55 (16.36%)	6/36 (16.67%)	*p* > 0.999
Endovascular coiling	69/91 (75.82%)	41/55 (74.55%)	28/36 (77.78%)	*p* = 0.806
No aneurysm-securing procedure	7/91 (7.69%)	5/55 (9.09%)	2/36 (5.56%)	*p* = 0.699
Ventricular drain (VD) insertion	52/115 (45.22%)	29/61(47.54%)	23/54 (42.59%)	*p* = 0.731
VD insertion within 72 h	49/115 (42.61%)	27/61 (44.26%)	22/54 (40.74%)	*p* = 0.848
Lumbal drain (LD) insertion	8/115 (6.96%)	5/61 (8.20%)	3/54 (5.56%)	*p* = 0.721
LD insertion within 72 h	5/115 (4.35%)	3/61 (4.92%)	2/54 (3.70%)	*p* = 0.890
Intraarterial spasmolysis	23/115 (20.00%)	13/61 (21.31%)	10/54 (18.52%)	*p* = 0.889
Intraasrterial spasmolysis ≥ 4 times	13/115 (11.30%)	8/61 (13.11%)	5/54 (9.26%)	*p* = 0.721

### Vitamin D status and the risk of vasospasm and cerebral ischemia

#### Cerebral vasospasm

Vasospasm could not be assessed in 30/115 patients due to the absence of an adequate acoustic window or early in-hospital death. Among the remaining 85 SAH patients, vasospasm occurred in 51/85 patients (60.00%). The occurrence of vasospasm did not significantly differ between study groups (VDD: 26/41, 63.41% vs. VDS: 25/44, 56.82%). These results suggest that vitamin D deficiency was not associated with a significantly increased risk of cerebral vasospasm in this cohort.

#### Novel ischaemic lesions on native CT imaging

**Fig. 2:**
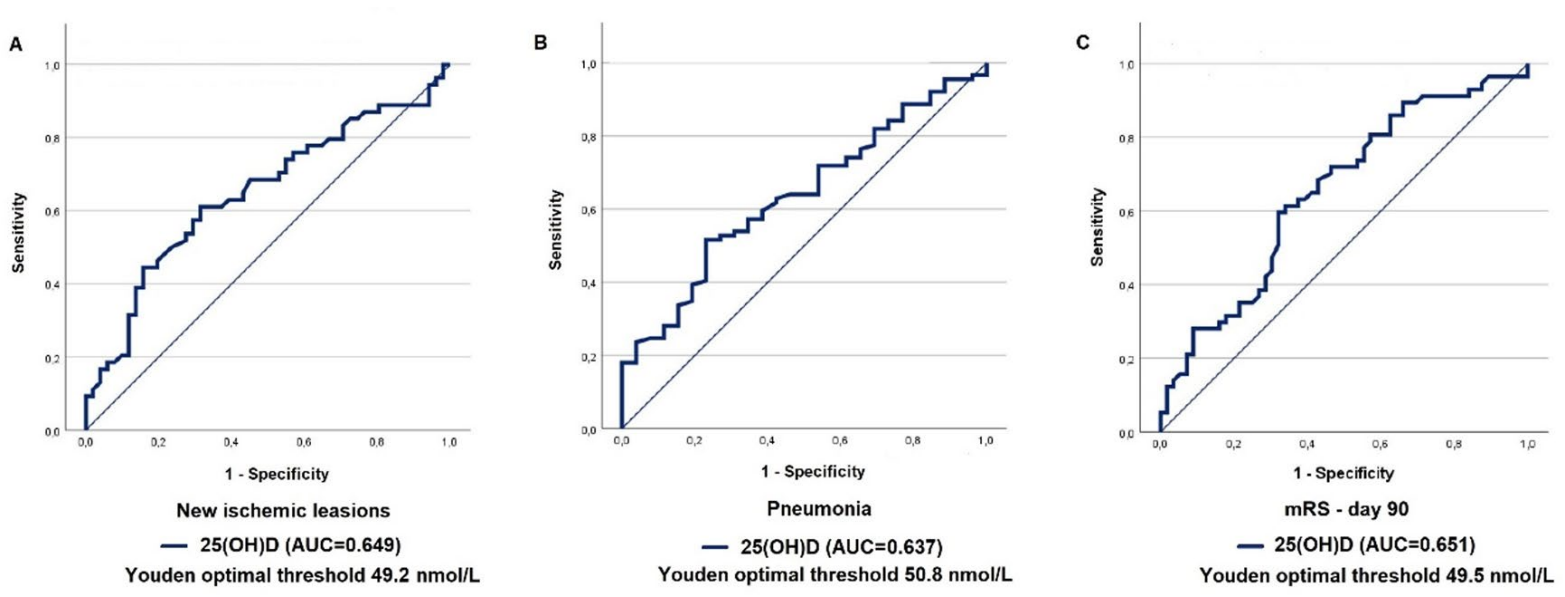
ROC curve analysis of serum 25(OH)D levels for the prediction of new ischemic lesions (**A**), pneumonia (**B**), and poor functional outcome according to mRS at 90 days (**C**). The analyses aimed to identify vitamin D thresholds associated with increased risk of complications and poor clinical outcomes. AUC values and optimal cut-off values determined by the Youden index are indicated below the plots. AUC: area under the curve. mRS: modified Rankin Scale. 25(OH)D: 25-hydroxy vitamin D.

Native cranial CT imaging was performed either in the case of clinical deterioration or as part of routine monitoring, following institutional protocols. New ischemic lesions, in comparison to the initial cranial CT scan performed at admission, were documented. Follow-up CT images were unavailable in 10 patients due to early mortality. The incidence of new ischemic lesions was significantly higher in the VDD group compared to the VDS group (35/51, 68.63% vs. 5/22, 40.74%, OR = 3.18; CI: [1.43; 7.1]; *p* = 0.008), indicating a potential association between vitamin D deficiency and increased risk of secondary ischemic injury. To evaluate the predictive value of vitamin D deficiency for new ischaemic lesions, ROC curve analysis was performed. The AUC was 0.649 (95% CI: [0.542; 0.755]), suggesting modest predictive value. The optimal cut-off vitamin D value based on the Youden index was 49.20 nmol/L, below this threshold, the risk of developing new ischemic lesions appeared to be notably elevated. (Fig. 2A)

#### Factors associated with the development of DCI

In univariable comparisons, DCI was more frequent among aneurysmal SAH patients (96.1% vs. 68.5%, *p* < 0.001), vitamin-D-deficient patients (68.6% vs. 40.7%, *p* = 0.008), and when norepinephrine treatment was used (74.5% vs. 37.0%, *p* < 0.001); other variables (age, smoking, modified Fisher score and GCS < 9) were non-significant (Supplemental Table 1.). In the multivariable logistic model, vitamin-D deficiency remained independently associated with DCI (OR 3.30, 95% CI 1.18–9.22; *p* = 0.023) together with norepinephrine use (OR 4.64, 95% CI 1.63–13.23; *p* = 0.004). Female sex showed a protective association (OR 0.32, 95% CI 0.10–0.99; *p* = 0.048).

### Association between inflammatory markers, infectious complications, and vitamin D status

#### Inflammatory markers

We calculated the median peak values of CRP (CRPmax), neutrophil to lymphocyte ratio (NLRmax), and interleukin-6 (IL6max) measured during the first 21 days after SAH.

**Fig. 3:**
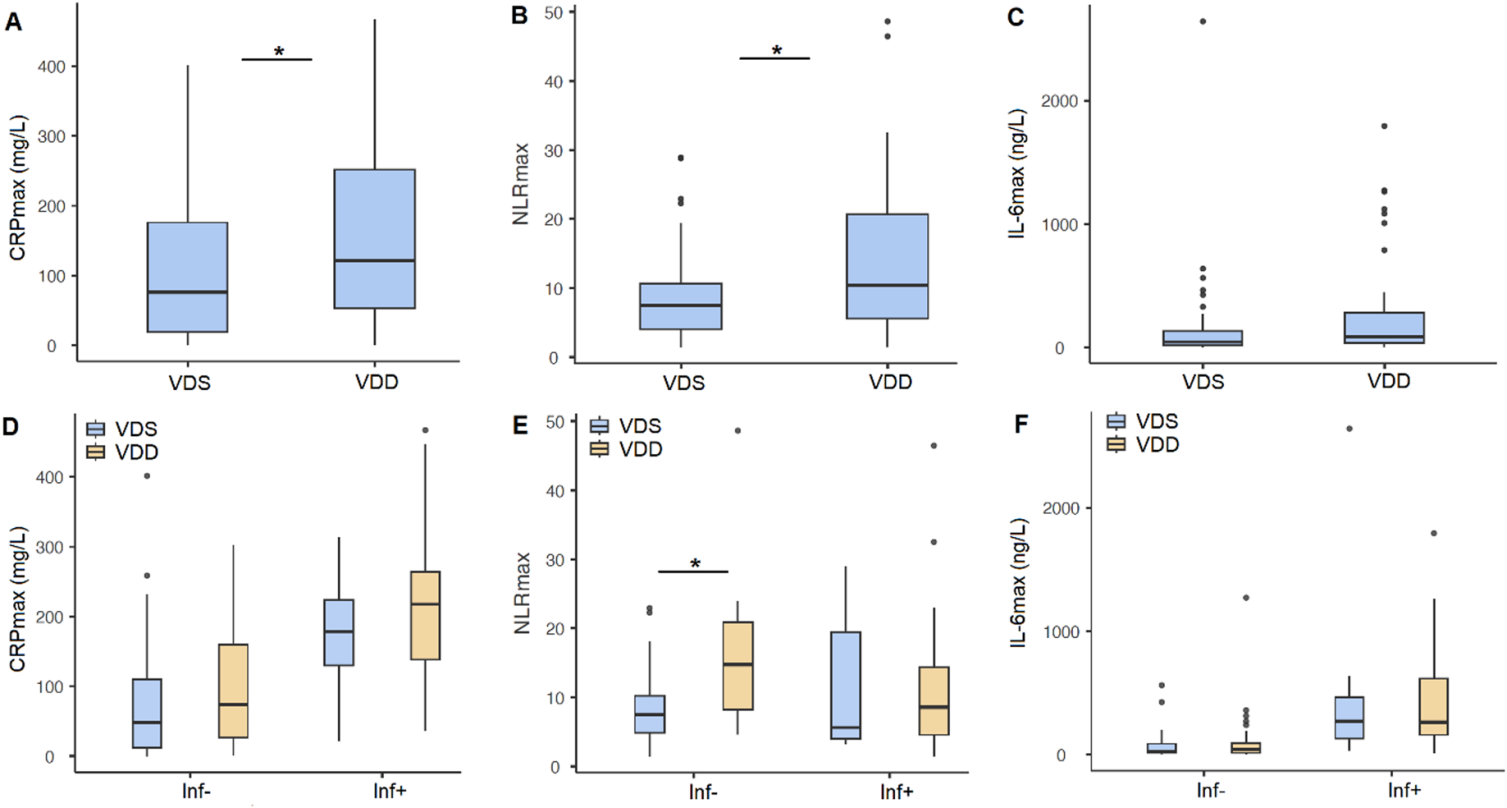
Inflammatory markers according to vitamin D status and presence of infectious complications. Panel A-C shows peak inflammatory marker levels in vitamin D sufficient (VDS) and vitamin D deficient (VDD) patient groups. Significantly higher CRP and NLR peaks were observed in the VDD group (*p* < 0.05). Panel D-F shows a stratified analysis of inflammatory markers according to infection status (Inf−: no infection; Inf+: infection present) and vitamin D status. A significant increase in NLR peak was found in VDD patients without infection (*p* < 0.05), suggesting infection-independent increased systemic inflammatory response. Boxplots show median, interquartile range, and outliers. CRPmax: peak C-reactive protein level. NLRmax: peak neutrophil-to-lymphocyte ratio. IL6max: peak interleukin-6 level.

In the total study population, CRPmax was significantly higher in the VDD group compared to the VDS group (121.75 mg/L [IQR: 53.39-251.91] vs. 76.26 mg/L [IQR: 18.91-176.16]; *p* = 0.018). Similarly, significantly higher NLRmax was confirmed in VDD patients versus VDS patients (10.38 [IQR: 5.59–20.65] vs. 7.48 [IQR: 4.03–10.6]; *p* = 0.034). IL6max also showed the trend, but the difference between the two groups did not reach statistical significance (84.76 ng/L [IQR: 35.46–281] vs. 43.6 ng/L [IQR: 16–132]; *p* = 0.066). (Fig. 3A-C)

Then, we further categorized our patients into infectious (Inf+) and non-infectious (Inf-) groups. In the Inf+ group, clinically significant infection was confirmed, based on he treating physician’s judgement, while patients in the Inf- group were free from infections. We investigated whether vitamin D status (VDD or VDS) within the Inf + and the Inf- group influences the elevation of inflammatory markers. In the Inf+ group, higher median CRPmax and IL6max and lower median NLRmax were found, compared to the Inf- group. However, vitamin D status (VDD or VDS) was not associated with significantly higher CRPmax or IL6max values within either the Inf + or Inf- groups. On the other hand, in the Inf- goup, VDD patients had significantly higher NLRmax values compared to VDS patients (13.9 [IQR: 8.2-20.86] vs. 7.52 [IQR: 4.9-10.21]; *p* = 0.006). (Fig. 3D-F)

These findings suggest that vitamin D deficiency may be associated with an overall enhanced inflammatory response following SAH. The disappearance of differences after further categorisation according to infectious state may be explained by a possible correlation between vitamin D deficiency and infectious complications. However, significantly elevated NLRmax among VDD patients in the Inf- group also suggests a partially infection-independent relationship.

#### Infectious complications

Infectious complications within the first 21 days after SAH were observed in 39/115 patients (33.91%). The VDD group had a higher incidence of infections (42.62%) compared to the VDS group (24.07%), however, the difference did not reach a statistically significant level. When the various types of infections were analysed separately, pneumonia has a significantly higher incidence in the VDD group (31.15% vs. 12.96%; *p* = 0.035; OR = 3.04, 95% CI [1.16–7.94]), while the incidence of other types of infections (bloodstream infection, ventriculitis/meningitis, urinary tract infection, and sepsis) did not show a statistically significant difference. (Table 3) ROC curve analysis was performed to determine the threshold serum vitamin D level under which the risk of pneumonia is notably increased. The area under the curve (AUC) was 0.637 (95% CI: 0.523–0.750). The Youden optimal threshold was 50.8 nmol/L, with a sensitivity of 73.1% and a specificity of 51.7%. (Fig. 2B).

**Table 3:** Incidence of infectious complications and their association with vitamin D status. VDD: vitamin D deficient group. VDS: vitamin D sufficient group. MDR: multidrug-resistant.

Parameters	Total Study Population (*n*/*N*, %)	Comparison of Study Groups Based on Vitamin-D Status			
		VDD (*n*/*N*, %)	VDS (*n*/*N*, %)	OR [95%CI]	*p*-Value (χ2)
Infection (unclassified)	39/115 (33.91%)	26/61 (42.62%)	13/54 (24.07%)	2.34 CI [1.05;5.24]	*p* = 0.057
Pneumonia	26/115 (22.61%)	19/61 (31.15%)	7/54 (12.96%)	3.04 CI [1.16;7.94]	*p* = 0.035^*^
Bloodstream Infection	14/115 (12.17%)	10/61 (16.39%)	4/54 (7.41%)	2.45 CI [0.72;8.33]	*p* = 0.164
Ventriculitis/ Meningits	6/115 (5.22%)	5/61 (8.2%)	1/54 (1.85%)	4.73 CI [0.54;41.85]	*p* = 0.212
Urinary Tract Infection	4/115 (3.47%)	1/61 (1.64%)	3/54 (5.56%)	0.28 CI [0.029;2.81]	*p* = 0.34
Sepsis (of any origin)	7/115 (6.09%)	5/61 (8.2%)	2/54 (37.04%)	2.32 CI [0.43;12.49]	*p* = 0.455
MDR bacteria	16/115	11/61	5/54	0.46 CI [0.15;1.43]	*p* = 0.191

### Functional outcome and mortality

Functional outcomes according to the modified Rankin scale (mRS) and Barthel’s Index (BI), as well as mortality on day 14, 30 and 90 were compared between VDD and VDS groups. The results of this analysis is shown on Table 4.

**Table 4:** Functional outcome and mortality at days 14, 30, and 90 in relation to vitamin D status. VDD: vitamin D deficient group. VDS: vitamin D sufficient group. mRS: modified Rankin scale. BI: Barthel index.

Outcome	Total Study Population (*n*/*N*, %)	Comparison of Study Groups Based on Vitamin-D Status			
		VDD (*n*/*N*, %)	VDS (*n*/*N*, %)	OR [95%CI]	*p*-value (χ^2^)
Day 14					
mRS > 2	82/113 (72.57%)	46/59 (77.97%)	36/54 (66.67%)	1.77 CI [0.77;4.08]	0.257
Mortality	19/113 (16.81%)	13/59 (22.03%)	6/54 (11.11%)	2.26 CI [0.79;6.45]	0.138
BI < 50	57/94 (60.64%)	32/46 (69.57%)	25/48 (52.08%)	2.1 CI [0.9;4.9]	0.128
Day 30					
mRS > 2	71/112 (63.39%)	43/58 (74.14%)	28/54 (51.85%)	2.66 CI [1.20;5.89]	0.024*
Mortality	23/112 (20.54%)	14/58 (24.14%)	9/54 (16.67%)	1.59 CI [0.62;4.05]	0.457
BI < 50	38/89 (42.7%)	25/44 (56.82%)	13/45 (28.89%)	3.24 CI [1.35;7.8]	0.014*
Day 90					
mRS > 2	55/113 (48.67%)	36/59 (61.02%)	19/54 (35.19%)	2.88 CI [1.34;6.2]	0.011*
Mortality	36/113 (31.86%)	23/59 (38.98%)	13/54 (24.07%)	2.01 CI [0.89;4.55]	0.134
BI < 50	12/77 (15.58%)	10/36 (27.78%)	2/41(4.88%)	7.5 CI [1.52;37.04]	0.01*

An mRS score of greater than 2 (moderate to severe disability or death) was more frequent in the VDD group at all follow-ups. The difference reached statistical significance on day 30 (74.14% vs. 51.85%, *p* = 0.024) and day 90 (61.02% vs. 35.19%, *p* = 0.011). To identify a vitamin D threshold that was associated with increased risk of poor functional outcome according to mRS on day 90, ROC analysis was performed. The area under the curve (AUC) was 0.651 (95% CI: 0.550–0.752). The Youden threshold for vitamin D was 49.5 nmol/L, with a sensitivity of 61.4% and a specificity of 64.3%. (Fig. 2C). We also performed a post hoc power analysis for the main outcome (VDD: 36/59 vs.19/54). The achieved statistical power was 0.78 at an alpha level of 0.05, indicating an approximately 78% probability of detecting significant differences between the groups.

Mortality rates were also higher in the VDD group at each follow-up point, although none of the differences reached statistical significance.

Among survivors BI < 50, indicating poor functional outcome, was more frequent in the VDD group, on days 14, 30, and 90. The difference reached the statistically significant level on days 30 and 90 (day 30: 56.82% vs. 28.89%, *p* = 0.014; day 90: 27.78% vs. 4.88%, *p* = 0.01).

To further investigate the association between vitamin D deficiency and unfavorable outcome (mRS > 2 at day 90), we employed a multivariate regression model including key confounders of the vitamin D - outcome relationship: aneurysmal subarachnoid hemorrhage, smoking, cerebral ischemic lesions, and pneumonia. In this analysis, new ischemic lesions (Odds Ratio = OR=2.86, [1.08; 7.6], *p* = 0.035) and pneumonia (OR = 8.0, [2.03; 31.49], *p* = 0.003) were associated with higher rates of mRS>2. In this model, the effect of vitamin D deficiency on outcome became non-significant, suggesting the possibility that the unfavourable effect of vitamin D deficiency on outcome may be at least partially mediated by other variables.

To explore this hypothesis, we performed causal mediation analyses. We tested whether the effect of vitamin D deficiency on poor 90-day outcome (according to mRS) was mediated by the development of new ischaemic lesions and pneumonia. The total effect (TE) of vitamin D deficiency on outcome was statistically significant (TE = 0.2560, *p* = 0.011 for the ischaemia model; TE = 0.2219, *p* = 0.014 for the pneumonia model), whereas the direct effect (ADE) was not (ADE = 0.1548 for ischaemia, *p* = 0.070; ADE = 0.1543 for pneumonia, *p* = 0.066). A trend toward a mediated (indirect) effect was observed through new ischaemic lesions (ACME = 0.1013; *p* = 0.058) and pneumonia (ACME = 0.0675; *p* = 0.069); however, neither reached statistical significance. These findings suggest a possible mediating role of these complications in the relationship between vitamin D deficiency and outcome, although the indirect effects did not reach statistical significance, possibly due to limited statistical power associated with the sample size.

We also tested whether seasonal variations exist in vitamin D levels in our cohort and whether this may have impact on outcome parameters. We arbitrarily dichotomised patients admitted in the period characterized with less sunshine (October-March) and more sunshine (April-September). Vitamin-D levels measured at admission in the „darker months” were significantly lower (mean ± SD: 45.6 ± 22.7 nmol/L) than in the „sunnier months” (mean ± SD: 57.5 ± 25.7 nmol/L, *p* = 0.01). Although vitamin D levels -treated as a continuous parameter- significantly differed between the two periods of the year, the difference between the numbers of VDD and VDS patients did not show any statistical significance (29/54 vs. 41/61, *p* = 0.14). There were no seasonal differences between mRS scores at 90 days (*p* = 0.97). Similarly, no significant season-related differences could be verified between the number of new ischemic lesions (31/61 vs. 20/44, *p* = 0.58).

## Discussion

This prospective observational study aimed to investigate whether vitamin D deficiency contributes to the development of certain neurological and non-neurological complications and worse clinical outcomes after spontaneous subarachnoid hemorrhage. The threshold for defining vitamin D- deficiency was based on Endocrine Society guideline^22^. During the 3-year enrollment period, 61 vitamin-D deficient (VDD, serum vitamin-D level < 50 nmol/L) patients have been diagnosed out of 115 acute non-traumatic SAH patients (53%). There was no statistically significant difference in demographic parameters, SAH severity scores, aneurysm location, comorbidities, and medication usage between the VDD and VDS study groups.

We observed that smoking and aneurysmal SAH were significantly more frequent among VDD patients compared to VDS patients. Smoking is a known independent risk factor for aneurysmal SAH^24^, but it is yet unknown whether vitamin D plays a mediating role in this pathophysiological pathway.

Vitamin D deficiency has been associated with intracranial aneurysm formation and rupture in multiple studies^25–27^. In our cohort, the prevalence of vitamin D deficiency (serum vitamin D level < 50 nmol/L) was 53%, which is higher than the 40.4% prevalence that has been reported in the average Hungarian population in a recent survey^28^. Among patients with a verified aneurysm, the prevalence of vitamin-D deficiency was even higher, 55/91 patients (60,4%), while among non-aneurysmal SAH patients, it was particularly low, 6/24 (25%). These findings support the previous hypothesis that vitamin D deficiency contributes to intracranial aneurysm rupture^25–27^. However, it is worth mentioning that a few studies highlighted that D-hypovitaminosis is very common among patients with acute diseases or injuries, and the level of 25(OH) vitamin D can decrease rapidly after a stressful insult. In the present study we sampled blood for Vitamin D measurements within 24 h after admission, right after the insult. Studies indicated that vitamin D behaves as a negative acute phase reactant. Thus, it can not be ruled out that values measured in acute conditions reflect rather disease severity, and D hypovitaminosis may be the consequence rather than the causative factor of acute illnesses ^14,16,29^. To put it more clearly: reverse causation cannot be excluded as low vitamin D-levels may also reflect the severity of the disease.

Among neurological complications, we studied the association between vitamin D status and the development of cerebral vasospasm and new cerebral ischemic lesions. Vitamin D affects poststroke neuroinflammatory responses, which play a critical role in the pathophysiology of ischemic lesions: it attenuates ischemic cell loss, regulates transcription of neuroprotective growth factors, contributing to CNS repair and shows vasodilatory properties^17,30^. In a mouse model of Kashefiolasl et al. found increased frequency of vasospasm during low vitamin D periods, such as wintertime and demonstrated a reduction in cerebral vasospasm, inflammation, and neurological deficit after 1,25(OH)vitamin D3 supplementation^31^. In a retrospective clinical study on 33 patients, Reyes et al. found no association between vasospasm and vitamin D status^32^. Prospective large-scale human studies are lacking in this field. Our study also failed to demonstrate an association between vitamin D status and cerebral vasospasm. However, in our cohort, the incidence of new ischaemic lesions was significantly higher in the VDD group compared to VDS, suggesting a vasospasm-independent neuroprotective effect of vitamin D. It should also be noted that VDD patients more frequently presented with aneurysmal SAH and were more often smokers, both of which are established risk factors for delayed cerebral ischemia (DCI). Assuming that the new ischemic lesions observed are consistent with DCI, this raises the question of whether VDD is truly associated with DCI, or whether the observed effect reflects baseline differences between groups. Lower serum vitamin-D levels in patients with ischemic stroke are independently associated with higher infarct volumes and higher scores on the National Institutes of Health Stroke Scale in several studies^33,34^. Human studies investigating the relationship between secondary ischemia after SAH and vitamin D status are lacking.

We also investigated whether vitamin D status affects the development of systemic inflammatory response and infectious complications. The hemorrhage activates the hypothalamo-pituitary-adrenal axis and induces a catecholamine surge, which promotes the production of chemokines and cytokines, resulting in a systemic inflammatory response^8,35^. Furthermore, it has been observed that not only the hemorrhage, but also the vasospasm and DCI can induce systemic inflammation^6^. As part of the systemic inflammatory response, a wide range of complications may develop, such as cardiac failure (Takotsubo cardiomyopathy), respiratory failure (neurogenic pulmonary oedema), acute kidney injury, cytokine release syndrome, anemia, and immunosuppression^35^. For the sake of describing the inflammatory state, we monitored the level of IL-6 and CRP, and we also calculated NLR at least every second day during the first 3 weeks after SAH. These three markers reflect different aspects of the inflammatory response, and according to previous studies, elevated levels predict worse outcome after SAH^36,37^. Our results showed that vitamin D deficiency was associated with higher average median CRP and NLR peak values, representing an overall enhanced inflammatory response following SAH. IL-6 did not show a significant correlation, which may be explained by its short half-life, potentially resulting in missing the real peak values in some cases. Elevated CRP and NLRpeak in the VDD group may reflect either increased non-infectious inflammatory response or increased rate of infection among vitamin D-deficient patients. In order to distinguish between infectious and non-infectious inflammatory responses, we further categorized our patients into infection-positive (Inf+) and infection-negative (Inf-) groups. Among Inf+ patients, vitamin D status did not significantly influence the levels of inflammatory markers. On the other hand, among Inf- patients average peak NLR was still significantly higher in the case of vitamin D deficiency, supporting a partially infection-independent enhanced inflammatory response. In our cohort, infectious complications, particularly pneumonia, were significantly more common among vitamin D-deficient patients, suggesting that vitamin D, besides modulating non-infectious immun response after SAH, also plays a role in the defence against pathogenic microorganisms. Although data in the SAH cohort are missing, it has to be noted that a recent meta-analysis of 7 randomised controlled trials showed that vitamin D supplementation reduces antibiotic utilisation in patients with respiratory tract infection, vitamin D insufficiency (vitamin D < 75 nmol/L), or aged below 70 years^38^.

Outcome measure scales (mRS, BI) indicated worse clinical outcomes among vitamin D-deficient patients in our cohort; however, mortality data did not show significant differences. Vitamin D may influence outcomes through several potential mechanisms. Analysis of our data suggests a secondary cerebral ischemia and pneumonia-mediated pathway. Besides this, according to previous studies, haematoma clearance and the development of post-stroke depression and cognitive impairment also impact outcomes and they may also be influenced by vitamin D levels^9,39,40^.

Based on ROC analysis of our data, using a threshold below 50 nmol/L 25(OH)vitamin-D serum concentration, the risk of new ischemic lesions, pneumonia, and poor 90-day outcome according to mRS is notably elevated. This threshold can serve as a target level for further interventional studies investigating the effect of vitamin D supplementation in SAH. Our findings raise the question whether vitamin D supplementation has the potential to decrease the risk of secondary brain ischemia, infectious complications and improve SAH outcome. Data from prior research on ischemic stroke and critically ill patients are promising; however, no randomized clinical trials are exist in the field of hemorrhagic stroke or SAH. In a recent meta-analysis of nineteen randomized controlled trials, involving critically ill adults, vitamin D supplementation significantly reduced short-term mortality, duration of mechanical ventilation, and length of ICU stay^41^. Prospective randomized controlled trials are needed to test the efficacy of vitamin D supplementation after SAH, to determine the optimal serum vitamin D target level, and the recommended route and dosage of supplementation.

Finally, we have to mention some limitations of our study. First, as a single-centre study, the number of enrolled patients is relatively low, limiting the statistical power of and the generalizability of our findings. Second, vasospasm was mainly diagnosed with TCCD, which has a limited sensitivity and specificity for detecting vasospasm in the anterior and posterior cerebral arteries. Lindegaard ratios were not assessed; only absolute blood flow velocities were regularly checked on a daily basis. Although this might be a further limitation, it has to be mentioned that the Lindegaard ratio modestly increases the sensitivity of vasospasm diagnosis^42^. Hence, daily transcranial Doppler measurements of cerebral blood flow velocities enabled a daily follow-up and recognition of cerebral vasospasm and contributed to the indication of cerebral angiography. Finally, it has to be emphasized that low vitamin D3 levels may represent a consequence of more severe injury, which could subsequently lead to complications and poorer prognosis, rather than hypovitaminosis itself being a causative factor.

## Conclusion

To the best of our knowledge, this is the first prospective study to assess the relationship between vitamin D status, complications, and clinical outcomes in patients with SAH. Our findings highlight the potentially harmful effects of vitamin D deficiency, 25 (OH)vitamin D serum level < 50 nmol/L, in SAH patients and may provide a basis for further clinical trials in this field. These trials should address different clinical questions, such as:

- Are low vitamin D levels measured at admission the consequence of SAH severity or whether pre-ictus hypovitaminosis also play a role in determining aneurysm rupture and the worse prognosis?
- Does supplementation of vitamin D contribute to better outcomes in patients with low vitamin D levels measured at admission in patients with aneurysmal subarachnoid hemorrhage?

## Supplementary Information

Below is the link to the electronic supplementary material.

Supplementary Material 1

Supplementary Material 2

## Data Availability

data will be available from the corresponding author after a reasonable request.
