# Supplementary material for: Impact of vitamin D deficiency on clinical outcomes in non-traumatic subarachnoid hemorrhage: A single-center prospective cohort study
Source: Sci Rep. 2026 Feb 4;16:7320. doi: 10.1038/s41598-026-38728-9 (PMC12923900; doi:10.1038/s41598-026-38728-9)
Supplement: Supplementary file 1 — Supplementary Material 1 [file 41598_2026_38728_MOESM1_ESM.docx]

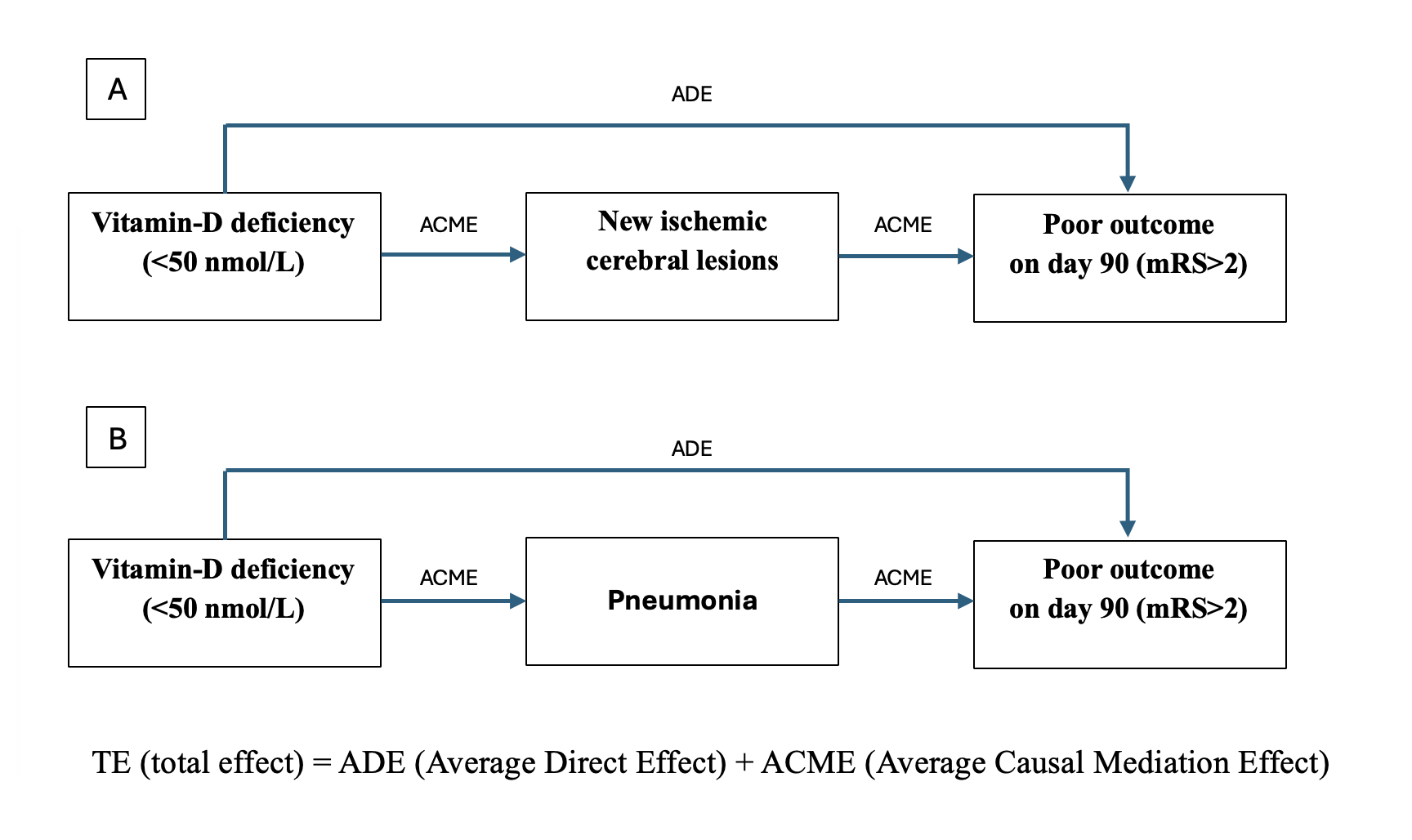


**Supplemental Figure 1.**Parallel single-mediator causal mediation models of the association between vitamin-D deficiency (<50 nmol/L) and poor 90-day outcome (mRS > 2). A) Mediation via new ischaemic cerebral lesions. B) Mediation via pneumonia. ADE = Average Direct Effect (exposure→outcome not through the mediator). ACME = Average Causal Mediation Effect (indirect effect along exposure→mediator→outcome). TE (total effect) = ADE + ACME.
