# Supplementary material for: Impact of vitamin D deficiency on clinical outcomes in non-traumatic subarachnoid hemorrhage: A single-center prospective cohort study
Source: Sci Rep. 2026 Feb 4;16:7320. doi: 10.1038/s41598-026-38728-9 (PMC12923900; doi:10.1038/s41598-026-38728-9)
Supplement: Supplementary file 2 — Supplementary Material 2 [file 41598_2026_38728_MOESM2_ESM.docx]

**Supplemental Table 1.** Multivariate analysis for the development of DCI

| Parameters | Total Population (CT results available)  (n/N, %) | Comparison of Groups Based on DCI | | |
| --- | --- | --- | --- | --- |
|  |  | **DCI (n/N, %)** | **non-DCI (n/N, %)** | **p-Value (Fisher/ χ^2^ /T-test)** |
| Number of patients (n) | 105 | 51/115 (48,57%) | 54/115 (51,43%) |  |
| Gender (F) | 65/105 | 31/51 (60,78%) | 34/54 (62,96%) | p=0.977 |
| Age (years) | 56.04 (±12.74) | 57.57 (±12.48) | 53.8 (±12.8) | p=0.13 |
| modified Fisher score (>2) | 79/105 (75.24%) | 43/51 (84.31%) | 36/54 (66.67%) | p=0.062 |
| IVH | 58/105 (55.24%) | 30/51 (58.82%) | 28/54 (51.85%) | p=0.602 |
| ICH | 26/105 (24.76%) | 15/51 (29.41%) | 11/54 (20.37%) | p=0.397 |
| Hunt-Hess score (>3) | 37/105 (35.24%) | 22/51 (43.14%) | 15/54 (27.78%) | p=0.149 |
| WFNS score (>3) | 42/105 (40.00%) | 25/51(49.02%) | 17/54 (31.48%) | p=0.102 |
| GCS (<9) | 35/105 (33.33%) | 22/51 (43.14%) | 13/54 (24.07%) | p=0.062 |
| Aneurysmal SAH | 86/105 (81.90%) | 49/51(96.08%) | 37/54 **(**68.52%) | **p<0.001*** |
| Smoking | 32/105 (30.48%) | 20/51 (39.22%) | 12/54 (22.22%) | p=0.093 |
| Surgical clipping | 14/86 (16.28%) | 8/49 (16.33%) | 6/37 (16.22%) | p>0.999 |
| Endovascular coiling | 65/86 (75.58%) | 38/49 (77.55%) | 27/37 (72.97%) | p=0.8 |
| Norepinephrine use | 58/105 (55.24%) | 38/51 (74.51%) | 20/54 (37.04%) | **p<0.001*** |
| Vitamin D deficiency | 57/105 (54.29%) | 35/51 (68.63%) | 22/54 (40.74%) | **p=0.008** |
